# Statistical Properties of Parasite Density Estimators in Malaria

**DOI:** 10.1371/journal.pone.0051987

**Published:** 2013-03-14

**Authors:** Imen Hammami, Grégory Nuel, André Garcia

**Affiliations:** 1 Department of Applied Mathematics (MAP5), UMR CNRS 8145, Paris Descartes University, Paris, France; 2 Department of Mother and Child in Relation to Tropical Infections, UMR 216, Institut de Recherche pour le Développement, Paris, France; Universidade Federal de Minas Gerais, Brazil

## Abstract

Malaria is a global health problem responsible for nearly one million deaths every year around 85% of which concern children younger than five years old in Sub-Saharan Africa. In addition, around 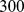 million clinical cases are declared every year. The level of infection, expressed as parasite density, is classically defined as the number of asexual parasites relative to a microliter of blood. Microscopy of Giemsa-stained thick blood films is the gold standard for parasite enumeration. Parasite density estimation methods usually involve threshold values; either the number of white blood cells counted or the number of high power fields read. However, the statistical properties of parasite density estimators generated by these methods have largely been overlooked.

Here, we studied the statistical properties (mean error, coefficient of variation, false negative rates) of parasite density estimators of commonly used threshold-based counting techniques depending on variable threshold values. We also assessed the influence of the thresholds on the cost-effectiveness of parasite density estimation methods. In addition, we gave more insights on the behavior of measurement errors according to varying threshold values, and on what should be the optimal threshold values that minimize this variability.

## Introduction

Malaria is caused by one or a combination of four species of *Plasmodia*: *Plasmodium falciparum*, *P. vivax*, *P. malariae*, and *P. ovale*. *P. falciparum* is the *Plasmodium* species responsible for 85% of the malaria cases and causes the most severe form of the disease. The three other species are less common and less dangerous. The usual and most reliable diagnosis of malaria is microscopic examination of blood films [1]–[6]. Two sorts of blood films can be used, thin or thick films, sampled from a finger prick. The thin smear is air-dried for 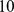 minutes. After drying, it is fixed in methanol and stained with Giemsa. Unlike the thin smear, the thick smear is dried for 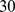 minutes, but not fixed with methanol. It is de-hemoglobinized in water, and then stained with Giemsa [7]. Thin films allow species identification, because the parasite's appearance is better preserved. Thick blood smears are most useful for detecting the presence of parasites because they allow examining a larger sample of blood [8], [9]. Thick films are hence more sensitive than thin films in case of low levels of infection and are therefore used to detect infection, and to estimate parasite density (PD) [1], [10], [11]. The level of infection, expressed as parasite density is classically defined as the number of asexual forms of parasite relative to a blood volume (e.g. microliter) or a percentage of white blood cells (WBCs).

Parasite density estimation methods usually involve threshold-based counting techniques [11]–[17]. Threshold definition and values may vary from one method to another. In some cases, parasites are counted in relation to the number of microscopic high power fields (HPFs), defined as oil immersion microscopic fields (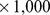), and in other cases parasites are counted according to a fixed number of leukocytes (WBCs). In the first cases, the methodology is: if less than 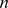 parasites are counted in the *m* first HPFs, then do this, else do that. In the second ones, the *m* first HPFs are replaced by “when 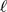 leukocytes are counted”, then do this, else do that. Conversion to counts per microliter then depends on the assumption that there is an average of 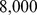 WBCs per microliter of blood.

Epidemiological interpretations must rely on solid evidence, hence the importance of reproducibility for parasite density data. However, all the methods used to determine PD potentially induce variability. To deal with this potential inaccurate estimation of PD, research teams tend to analyze more slides and subjects. By taking duplicate readings or larger sample sizes, we can statistically improve our knowledge of the PD being measured. Then, we can decrease the variability in microscope slide readings and improve the accuracy and reproducibility of the measurements. However, one of the problems the research teams have to deal with is that during large scale studies the number of thick blood smears performed can be greater than 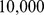. Then, the repetition of the microscope slide examination leads to an important cost overrun in terms of both money and time. One may wonder whether such practices have a significant interest for the final results. With low parasitemias, it is probably worth the effort of reading more slides. But in some situations it is not needed, for example, with large parasitemias levels.

To our knowledge, none of the studies of variability have dealt with the sampling error generated by the threshold-based counting techniques or evaluated the impact of the existing threshold values in endpoint measurements. In addition, the accuracy and consistency of these methods have largely been overlooked. Furthermore, there is no general agreement on the optimal method for estimating parasite density according to threshold values. Further experimental evidence is needed to determine which parasite counting technique is most accurate, reproducible, and efficient. The aim of this paper was to explore the variability of four frequently used threshold-based counting methods of determination of PD. For each of these methods, we assessed the consequences that a modification of the threshold can have on variability.

## Materials and Methods

### Threshold-based counting techniques

PD estimates accuracy vary significantly depending on the methodology from which they are derived. The estimation method differs from one health care organisation to another. To summarize, there are four basic types of threshold-based counting techniques commonly used in epidemiological surveys.

Dowling & Shute (1966) [12] propose to count parasites in 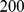 consecutive HPFs in thick blood film [7], [18]. They consider that the volume of blood corresponding to 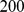 HPF of a thick film is 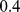 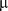
l, and that examining 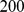 HPFs is the best compromise between the need to reduce the risk of missing parasites and the need to minimize the reading time. This method will be referred to as Method A. Here, the number of HPFs read is the threshold value. We investigate the influence of this number on the reliability of the PD estimation.

Trape (1985) [11] proposes to count the total number of parasites per 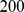 WBCs. The average value of 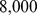 WBCs per 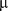
l is accepted as reasonably accurate by *The World Health Organization* (WHO) [17]. Then, the number of parasites counted is multiplied by 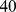 to give the number of parasites per 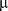
l [9], [19], [20]. This method will be referred to as Method B. In this method, only one threshold value is specified, which is the number of leukocytes seen 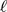. We are interested in how the value of 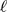 affects measures of variability.

According to the WHO recommendations [17], the number of parasites should be counted on one tally counter and the number of white blood cells should be counted on a second one. The number of parasites and white blood cells counted depend on how numerous the parasites are. The lower the number of parasites counted, the higher the number of WBCs that should be counted. Parasites are counted until 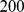 WBCs have been seen. If 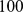 or more parasites are found, the number of parasites per 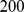 WBCs is then recorded. Else, counting should be continued up to 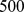 WBCs. This method will be referred to as Method C. Three parameters are specified : the required number of parasites 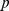, the required number of leukocytes in the first step 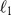, and the required number of leukocytes in the second step 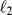. Modeling, estimating and validating multidimensional distribution functions cast difficult problems, both conceptual and technical. For that reason, it is more convenient to fix 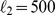 and to study the method's performance by varying the two parameters 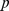 and 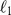. Hence, we obtain the influence of adding an extra threshold value on the final estimation.

Finally, the last method presented in this paper was used during a research program conducted in the *Tori Bossito* area in *Southern Benin*. In this program, the PD is determined by simultaneously counting parasites and leukocytes. The counting stops when either 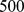 WBCs or 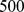 parasites are seen whichever comes first [15]. This method will be referred to as method D. Two parameters are specified : the required number of parasites 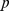 and the required number of leukocytes 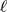. We analyze the performance of the method with respect to effectiveness and efficiency for different values of parameters 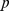 and 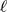.

Unlike methods A and B, methods C and D are adaptative methods. In these methods, counting stops when parasites are found in sufficient number. Hence, their cost is reduced for high parasitemias.

### Measures of variability

The source and scale of measurement error depends on several parameters, such as sample preparation, staining process, counting technique, microscopist performance, etc. However, variation of parasite density within a slide is expected even when prepared from a homogeneous sample [21]. The sampling variability is a source of interest when studying the efficiency of estimation methods. It refers to the different values which a given function of the data takes when it is computed for two or more samples drawn from the same population. In this paper we are interested in the sampling errors and biases induced by threshold-based counting techniques and more particularly in the impact of threshold values in endpoint measurements.

Let 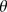 be the parameter that denotes the real value of the PD per microliter of blood and let 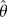 be its estimate. Since 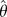 is a random variable, it can never been said with certainty that this estimate is close to the true value of 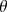. For that reason, we consider its statistical properties, that is, its probability distribution 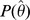, or some restricted aspects thereof. Here, we focuss on variability measures : mean error (ME), coefficient of variation (CV) and false negative rate (FNR).

#### Mean error

In order to define the variability measures, we need to introduce the concept of mathematical expectation. The expected value of the estimator 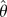 denoted as 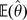 is an average taken over all possible values of 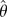. Suppose 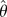 takes value 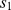 with probability 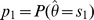, value 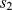 with probability 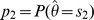, and so on, up to value 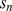 with probability 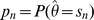. Then the expectation of 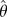 is defined as
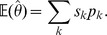



The sampling bias occurs when the true value (in the population) differs from the observed value (in the study) due to a flaw in the sample selection process. An estimator *bias* is the difference between the estimator's expected value 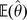 and the true value of the estimated parameter 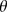. Hence, in computing the bias induced by different counting techniques, we used 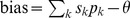. An estimator with zero bias is called *unbiased*. Mean error is the bias expressed as a percentage of 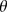, i.e. 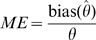. It provides a measure of the magnitude of the bias and allows comparing different methods.

#### Coefficient of variation

A measure of the sampling error is the standard deviation which is the square root of its variance. Standard deviation is a measure of dispersion from the mean, or the expected value and it is commonly used to compute confidence intervals in statistical inferences. The reported margin of error is typically about twice the standard deviation (1.96), the radius of a 95 percent confidence interval. Sampling variability can also be expressed relative to the estimate itself through the coefficient of variation (CV), which is defined as the ratio of the standard deviation 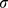 to the true value 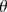. In computing the CV induced by different estimation methods, we used
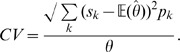



Then, CV is expressed as a percentage of 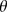.

#### False negative rate

The last measure of variability we study is the false negative rate (FNR), also known as Type II error or 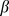 error, which is the error of failing to reject a false null hypothesis. The false negative rate indicates the probability of a counting method to estimate PD as null when it is not, i.e. 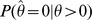.

#### Cost

In addition to the variability, we are also interested in the cost-effectiveness of each method. We define the method's cost as the number of HPFs that has to be read to reach the threshold value. Once it is reached, we stop the examination of the smear. Depending on the method being used, the cost is based on the number of parasites (or WBCs) required to stop the reading of the thick blood smear. Let T denote the required number of HPFs to stop the counting. We first compute 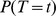. Then, we express the Method cost as 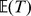.

### Methodology

The following methodology is used to compute the three measures of variability (ME, CV and FNR) and to assess cost-effectiveness of methods. Firstly, the exact distribution of 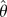 is computed through recursive formulas. Secondly, based on this probability density function, measures of variability are derived. Finally, cost-effectiveness is defined for each method as the required number of HPFs that has to be read until the threshold is reached. A C++ program is used to implement these recursive formulas. The calculations are performed under two assumptions :

A1. The distribution of the thickness of the smear, and hence of the parasites within the smear, is homogeneous.

A2. The distribution of the parasites in the HPFs is uniform, and thus can be modeled through a Poisson distribution [21]–[23].

#### Notations

Let 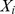 be a random variable that represents the number of parasites in the 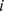-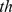 HPF. Suppose that 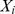 are independent and identically distributed (i.i.d.).

Under an assumption of uniformity, the number of parasites per field can be modeled using Poisson distribution (assumption A2). If the expected number of parasites per HPF is 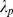, then 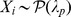. Thus 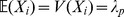.

Let 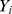 be a random variable that represents the number of leukocytes in the 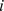 *-* 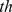 HPF. Suppose that 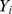 are independent and identically distributed (*i.i.d.*). Leukocytes are supposed evenly distributed over the thick smear. Therefore, the number of leukocytes per field can be modeled using the Poisson distribution. If the expected number of parasites per HPF is 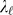, then 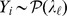. Thus, 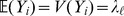.

- Let 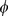 denote the parasite density per WBC.
- Let 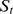 be the sum of parasites in 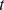 consecutive HPFs.
  Then, 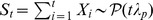.
- Let 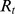 be the sum of leukocytes in 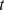 consecutive HPFs.
  Then, 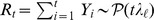.
- Let 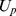 be the minimum number of HPFs required to obtain 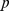 parasites.

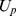
 can be expressed in terms of 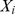 as follows
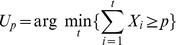

  Probability of 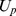 is given by
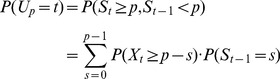
(1)
- Let 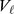 be the minimum number of HPFs required to obtain 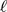 leukocytes.

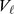
 can be expressed in terms of 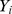 as follows
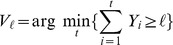

  The probability mass function of 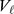 is given by
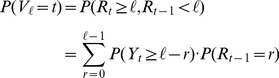
(2)

#### PD estimation

For method A, natural estimator of 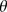 is used and the exacts formulas of ME, CV and FNR are given. However, the estimation of 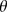 is not straightforward for the remaining methods (B, C, D). Hence, recurrence formulas are used to derive variability measures.

Let 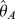 be the estimator of 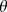 for Method A. Let 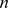 be the number of HPF read. Since 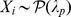 and 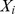 are iid, we have 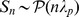. The number of parasite per field 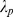 is then estimated by 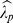 where 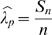. Assuming the average amount of blood in each field as 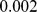 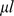 [12], the PD is estimated by 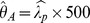. Since 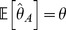, 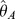 is *unbiased*. Thus, the ME is null.

In order to evaluate the efficiency of this estimator, the variance is to be compared against the Fisher Information I(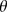). The variance of this unbiased estimator is bounded by the inverse of the I(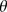); namely the *Cramer-Rao* Bound (CRB). We show that the variance of the proposed estimation technique reaches the Cramer-Rao lower bound. Hence, 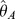 is an efficient estimator of 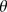 (the proof is given in Section 1 in File S1).

The coefficient of variation (CV) is defined as the ratio of the standard deviation 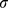 to 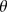, which is equal to 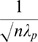.

In practice, false negatives occur when diagnosing by mistake PD as null after reading 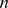 HPFs, i.e. 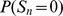, which gives 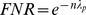.

For Method B, 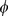 is estimated by 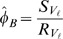. Then, 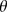 is estimated by 
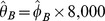
.

To derive statistical properties of the PD estimate, we first need to compute the probability of seeing 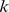 parasites (resp. 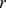 leukocytes) in 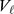 HPFs. These probabilities can be expressed as follows
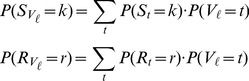
(3)



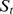
 and 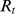 are Poisson-distributed and the probability of 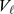 is computed according to Equation (2).

The probability density function of 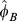 is
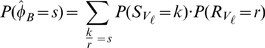



Let 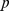 be the required number of parasites for Method C. Let 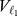 be the minimum number of HPF required to obtain 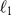 leukocytes and 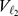 be the minimum number of HPF required to obtain 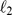 leukocytes. Let 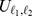 be the minimum number of HPFs required to obtain 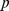 parasites. The probability mass function of 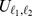 is as follows
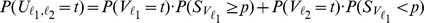



Probabilities of 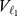 and 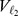 are computed according to Equation (2).

Probability of 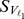 is computed according to Equation (3).

Then, 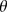 is estimated as follows 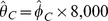, where 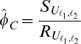.

The probability density function of 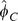 is
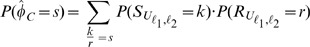



For Method D, 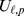 denotes the minimum number of HPF required to obtain either 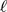 leukocytes or 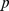 parasites, i.e. 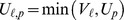. The probability mass function of 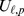 is as follows
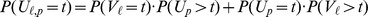



Probabilities of 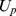 and 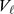 are computed according to Equation (1) and Equation (2).

Then, 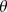 is estimated by 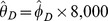, where 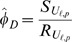.

The probability density function of 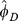 is
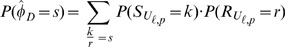



#### Validation study

Simulations are used to study the accuracy of our mathematical models, and to validate the theoretical results derived from estimators' probability functions. For the purpose of simulations, we predefine a data-generating model of 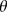. Given 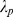 and 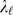, PD data are sequentially generated for each HPF. In that way, random samples of 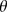 are generated under the Poisson assumption. Then, we investigate properties of sample means, variances and FNR. We use the statistical software package R to perform 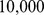 simulations. In each simulation step, we generate 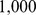 random drawings of 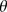 and we save the sample ME, CV, FNR and cost in a vector. In that way, we were able to investigate the results of all simulation steps. We compare the simulated results to the theoretical ones. Some results of our validation study are given in Section 2 in File S1. Simulations are computationally expensive. Then, it is burdensome to have to perform 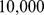 simulations to estimate each PD value according to methods A, B, C and D. Hence, computing the exact distribution of 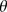 is a most useful alternative. Codes used for generating the data will be provided to the reader upon request.

#### Colormaps

The recursive formulas described above are used to compute the exact distribution of variables. Each computation takes as input: 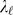 the number of leukocytes per field, 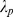 the number of parasites per field and the threshold values (number of HPFs, number of WBCs, number of parasites). The outputs are ME, CV, FNR and cost values. This approach is computationally expensive due to recursive formulas that precisely compute probability of getting 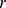 WBCs (resp. 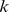 parasites) according to each counting technique. These probabilities are used to compute 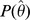. Statistical properties of PD estimators are then derived. These data sets are gridded into colormaps where the values taken by a variable (ME, CV, FNR, cost) in a two-dimensional table (X,Y) are represented as colors. Each rectangle in this grid is a pixel (or a color sample). This program sets each pixel to a color index according to its coordinates. Each pixel has an X and Y position where the X coordinate is the parasite density value and the Y coordinate is the threshold value. The X axis spans the range of 0 to 20,000 parasite per 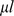 (400 values). The Y axis ranges from 0 to 500 (500 values). Hence, we used a resolution of 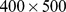 pixels. Contour lines are overlaid over the colormaps. A contour line connects points where the function has constant value. Linear interpolation is used in generating contour data. A higher resolution is needed to achieve a smoother mapping and to avoid artifacts (jagged contours), which arise due to interpolation.

We believe that if data mappings are addressed simultaneously in a single framework, the resulting approach will facilitate visual comparisons of methods. At that point, we consider the problem of scale. The methods use either different numbers of arguments or different types of arguments. We got rid of this by expressing variability measures for all methods as functions of parasity density and WBCs count. To do so, we convert threshold values used in each method into WBCs count. For Method A, we assume an average of 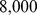 WBCs per microliter of blood [17] and an average of 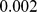 microliter of blood in each field [12]. The number of HPFs read 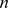 is then multiplied by 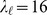 WBCs to give the number of WBCs counted in 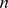 HPFs. For Method B, the thresold value is the number of WBCs counted 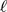. For Method C, we consider the case where 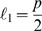 and we fix 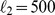. For Method D, we consider equal numbers of parasites 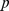 and leukocytes 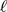 that have to be seen to stop the counting, hence 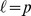. Theses decisions are based on common use and can be considered reasonable assumptions. Moreover, this two-dimensional representation has the conceptual advantage of reducing the number of arguments and ensures a common approach for assessing methods performance.

## Results

In the following, parasitemia was categorized as either low (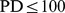 parasites/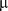
l), intermediate (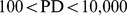 parasites/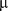
l) or high (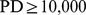 parasites/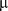
l).

Method C involves three threshold values, which leads to a multidimensional problem. For this reason, we chose to express the parasite count in the first step as the half of the leukocyte count. We fixed the leukocyte count in the second step to 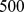 WBCs.

### Impact of thresholds on variability measures

#### Mean error

The mean value of the Method A estimator equals the true value of the PD. Therefore this estimator is called an unbiased estimator. In addition to having the lowest variance among unbiased estimators (so called Minimum Variance Unbiased Estimator), this estimator also satisfies the Cramér-Rao bound, which is an absolute lower bound on variance for statistics of a variable and thus is an unbiased efficient estimator. Analytic proofs are given in Section 1 in File S1.

**Figure 1:**
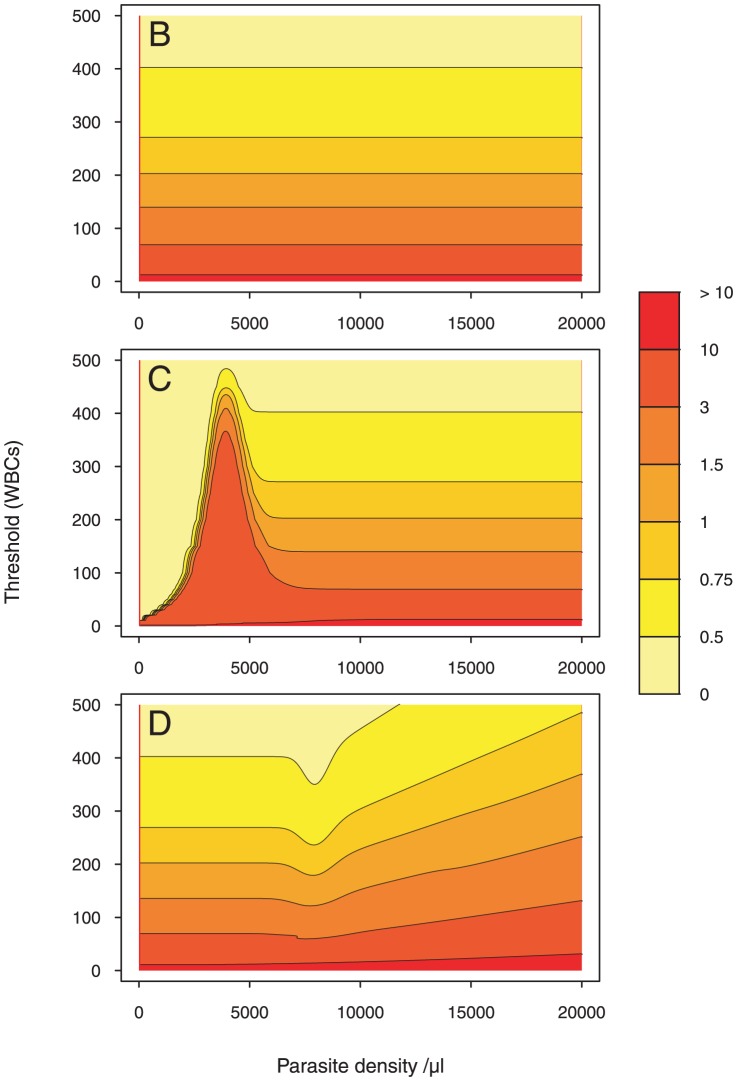
Mean error colormap. The colormap is drawn given a two-dimensional array of ME values. To allow for direct point-to-point numerical and visual comparison, we express the ME as a function of the parasite density (on the 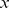-axis) and the WBC count (on the 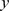-axis) in each of the four methods. Parasite density values are generated starting with 0, at increments of 50, and ending with 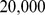. Threshold values (WBCs) are generated starting with 0, at increments of 1, and ending with 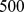. Then, each pixel is assigned a value that represents the ME-level. A color scale grading was applied to show levels. 7 degree intervals are depicted using a red-to-yellow colorspace with increasing intensity. We contour the ME at 0.5, 0.75, 1, 1.5, 3 and 10. The gaps between each pair of neighboring contour lines is filled with a color.

As shown in Figure 1, the ME of Method B only depends on threshold values. In Method B, parasites are counted until a fixed number of WBCs are seen and the number of parasites seen is not involved in the stopping rule of this counting process. Hence, the ME is independent from the PD. The colormap of the ME shows that the mean error decreases as threshold values increase. For instance, counting parasites until 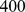 WBCs instead of 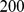 WBCs decreases the bias by 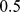
% of the parasite density. This can help choose the threshold value that allows to decrease the bias to a reasonable value.

For Method C, three parts can be distinguished. For 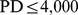 parasites/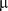
l, contour lines are increasing functions of the PD and the thresholds. The darkest part on the map represents a constant ME. Due to the limited number of parasites in this area, counting is carried out until 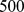 WBCs are seen. Hence, Method C has similar behavior to Method B for WBCs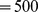. By counting up to 500 WBCs, the mean error is fixed to 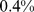. For 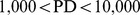 parasites/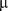
l, ME values are represented by a set of bell-shaped density curves with a peak reached at 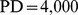 parasites/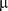
l. 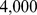 is half the standard number of WBCs per 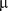
l. For Method C, the number of parasites counted is half the number of leukocytes. For 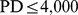 parasites/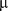
l, increasing the parasite density increases the leukocyte count for a fixed ME value. If the microscopist wants to estimate for constant ME a higher parasite density, he needs to count more leukocytes. A higher threshold value is then required due to the small number of parasites present in this area. For 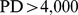, lower leukocyte counts are needed to maintain a constant ME value. A steady state will be reached afterwards whereby the ME is density independent. Due to the abundance of parasites, the ME only depends on the WBCs count. This steady-state region starts at 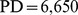 parasites/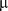
l for 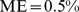.

Note that the same ME level may be reached by more than one threshold value (eg. two contour lines for 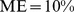).

For 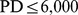 parasites/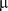
l, the ME generated by Method D is density independent (see Figure 1). In this interval, leukocytes are more abundant than parasites. Hence, parasites are counted until a predetermined number of leukocytes is reached. We notice that increasing the leukocyte count will not significantly reduce the bias. For instance, counting parasites until 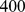 WBCs are seen, instead of 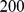, decreases the bias only by 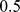
% of the parasite density. For 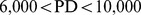 parasites/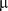
l, contour lines reach their minimum at 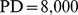 parasites/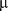
l. In this area, the number of leukocytes per field (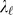) and the number of parasites per field (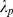) are very close. Lower threshold values are needed to maintain a constant ME. For high parasitemia, parasites are more numerous than leukocytes. The ME is therefore density dependent. If the microscopist wants to estimate for constant ME a higher parasite density, he needs to count more parasites.

#### Coefficient of variation

**Figure 2:**
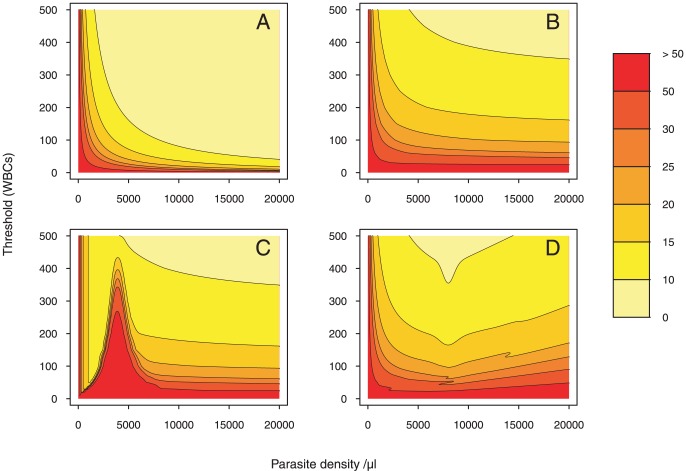
Coefficient of variation colormap. The colormap is drawn given a two-dimensional array of CV values. To allow for direct point-to-point numerical and visual comparison, we express the CV as a function of the parasite density (on the 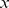-axis) and the WBC count (on the 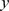-axis) in each of the four methods. Parasite density values are generated starting with 0, at increments of 50, and ending with 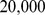. Threshold values (WBCs) are generated starting with 0, at increments of 1, and ending with 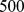. Then, each pixel is assigned a value that represents the CV-level. A color scale grading was applied to show levels. 7 degree intervals are depicted using a red-to-yellow colorspace with increasing intensity. We contour the CV at 10, 15, 20, 25, 30 and 50. The gaps between each pair of neighboring contour lines is filled with a color.

The CV of Method A is the inverse square root of 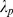 times the number of fields (see Materials & Methods). If the counting does not exceed 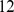 HPFs (i.e. 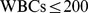), CV values are higher than 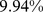 of the real PD for low and intermediate parasitemias (see Figure 2). Above 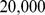 parasites/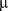
l, CV values are less than 3%. Counting up to 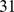 HPFs (i.e. 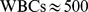) instead of 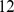 HPFs (i.e. 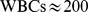), decreases the CV by approximately 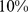 of the parasite density.

For Method B, CV values lie midway between 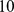
% and 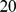
% of the PD for high parasitemias when 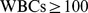 (see Figure 2). Notice that increasing the number of WBCs counted will not significantly decrease the CV for high parasitemias.

For Method C, the vertical lines indicate that the CV only depends on PD for low parasitemias. Due to the small number of parasites, CV levels are obtained by counting parasites until 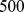 WBCs are seen. Notice that CV values exactly match those obtained by Method B with 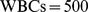. Figure 2 shows bell-shaped patterns for higher densities (for 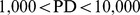 parasites/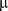
l) with a peak reached at 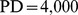 parasites/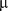
l. Along the same line as Method B, a constant CV level may be reached by more than one threshold value for 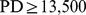 parasites/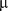
l.

For Method D, the negative slope of the contour lines captures the indirect relationship between the threshold and the densities for 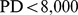 parasites/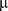
l. For a fixed CV level, threshold values decline with density. In this interval, the counting stops when the fixed number of leukocytes (i.e: the threshold value) is obtained. The minimum is reached at 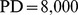 parasites/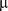
l. For 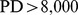 parasites/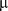
l, positively sloped CV curves reflect the direct relationship between the threshold and the PD. In this area, parasites are more abundant than leukocytes. Therefore, the counting stops when the fixed number of parasites (i.e: the threshold value) is reached. If the microscopist wants to estimate with the same level of precision (i.e: for constant CV) a higher parasite density, he needs to count more parasites. A higher threshold value is then required.

#### False negative rates

**Figure 3:**
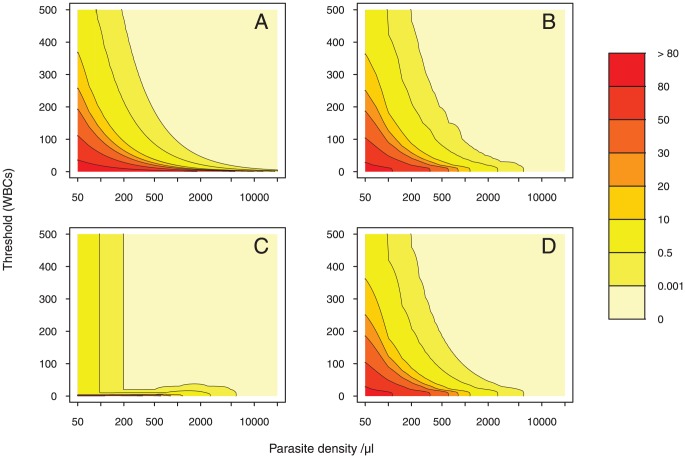
False negative rates colormap. The colormap is drawn given a two-dimensional array of FNR values. To allow for direct point-to-point numerical and visual comparison, we express the FNR as a function of the parasite density (on the 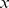-axis) and the WBC count (on the 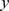-axis) in each of the four methods. Parasite density values are generated starting with 0, at increments of 50, and ending with 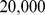. Threshold values (WBCs) are generated starting with 0, at increments of 1, and ending with 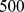. Then, each pixel is assigned a value that represents the FNR-level. A color scale grading was applied to show levels. 8 degree intervals are depicted using a red-to-yellow colorspace with increasing intensity. We contour the CV at 0.001, 0.5, 10, 20, 30, 50 and 80. The gaps between each pair of neighboring contour lines is filled with a color. A logarithmic scale is used on the 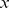-axis and a linear scale is used on the 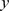-axis.

For Method A, FNR decreases exponentially with increasing number of fields (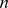) and increasing number of parasites per field (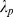) (see Materials & Methods). If the counting does not exceed 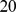 HPFs (i.e. 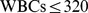), the probability of misdiagnosis is high for low parasitemia levels (see Figure 3). For intermediate densities, this probability is less than 1% when the threshold is above 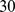 HPFs. For high parasitemias, false negatives occur much less frequently (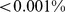). Despite unbiasedness and efficiency, this estimator generates a high number of false negatives when the problem is difficult (low parasitemia).

Figure 3 shows that the FNRs of Method B vary from 5% to 80% for low parasitemia levels. For intermediate densities, this probability is less than 5%. False negatives do not occur for high parasitemia levels.

For Method C, the FNRs are threshold independent for 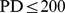 parasites/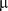
l and 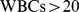. The number of false negatives arises from counting up to 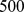 WBCs. For 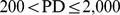 parasites/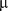
l and 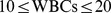, FNR values varies from 0.001% to 0.5%. For 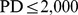 parasites/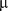
l and 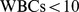, FNR values are higher than 0.5%. False negatives do not occur for high parasitemia levels (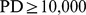 parasites/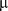
l).

For low parasitemias, we point out striking similarities between the FNRs in Method D and the FNRs in Method B. Due to the scarcity of parasites in this area, estimates are based on the leukocyte count in Method D.

#### Cost-effectiveness

Method A does not adapt to the variation of PD from one individual to another and costs a fixed HPFs number for all PD values.

**Figure 4:**
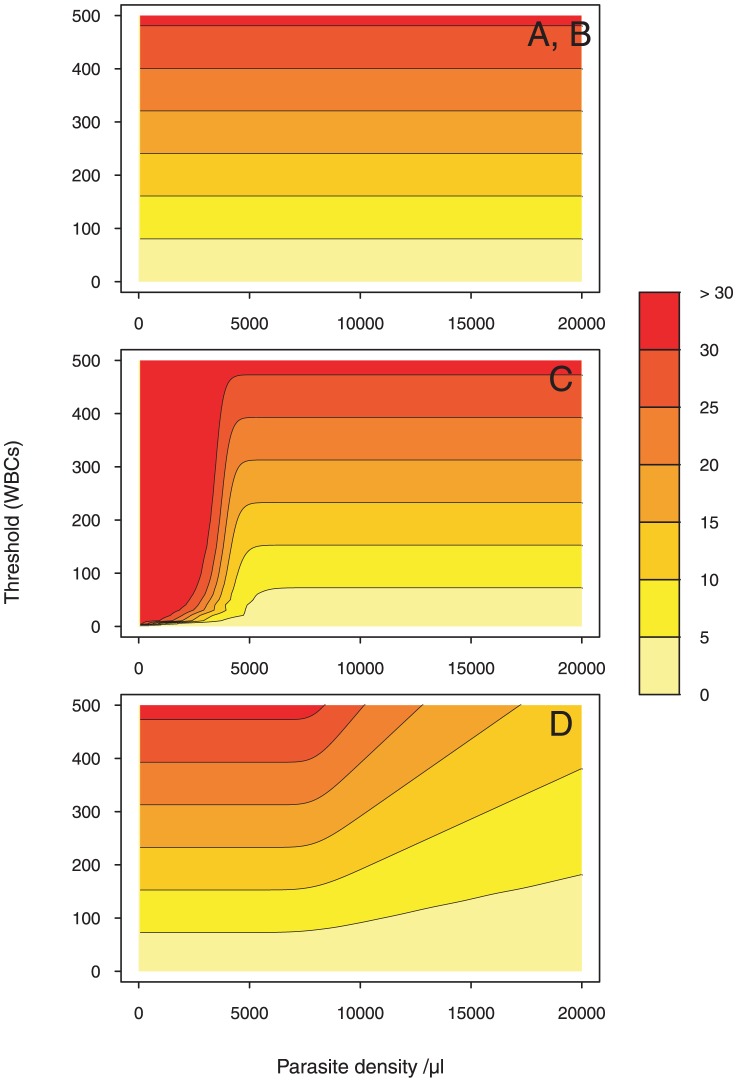
Cost-effectiveness colormap. The colormap is drawn given a two-dimensional array of cost values. To allow for direct point-to-point numerical and visual comparison, we express the cost as a function of the parasite density (on the 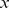-axis) and the WBC count (on the 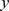-axis) in each of the four methods. Parasite density values are generated starting with 0, at increments of 50, and ending with 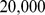. Threshold values (WBCs) are generated starting with 0, at increments of 1, and ending with 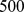. Then, each pixel is assigned a value that represents the cost-level. A color scale grading was applied to show levels. 7 degree intervals are depicted using a red-to-yellow colorspace with increasing intensity. We contour the cost at 5, 10, 15, 20, 25 and 30. The gaps between each pair of neighboring contour lines is filled with a color.

The cost of Method B is an increasing linear function of the threshold values (see Figure 4). The cost here is independent from PD. This can be explained by the homogeneous distribution of leukocytes within the fields. Since we assumed a fixed number of leukocytes per field (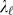), the number of fields needed will indeed be independent of the PD.

For low parasitemia levels and 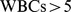, the darkest color in the cost colormap of Method C indicates a constant cost of approximately 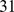 HPFs, which corresponds to the number of fields needed to reach 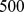 WBCs. For intermediate parasitemia levels, the cost varies depending on both the threshold value and how numerous the parasites are. The cost is independent from PD for high parasitemia levels. The number of fields needed is the ratio of WBCs to 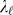.

Method D is highly adapted to parasitemia levels in terms of cost. For 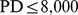 parasites/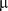
l, the cost is density independent. For 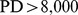 parasites/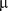
l, the cost decreases with density for a fixed threshold value. In this interval, parasites are more abundant than leukocytes. A lower number of fields is then needed to reach a predetermined threshold value.

### Methods comparison for three parasitemia levels

To explore similarities and differences in method behaviors, we look more closely at the statistical properties of PD estimates. We choose three cut-offs for low (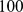 parasites/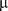
l), intermediate (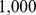 parasites/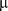
l) and high (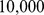 parasites/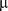
l) parasitemias.

**Figure 5:**
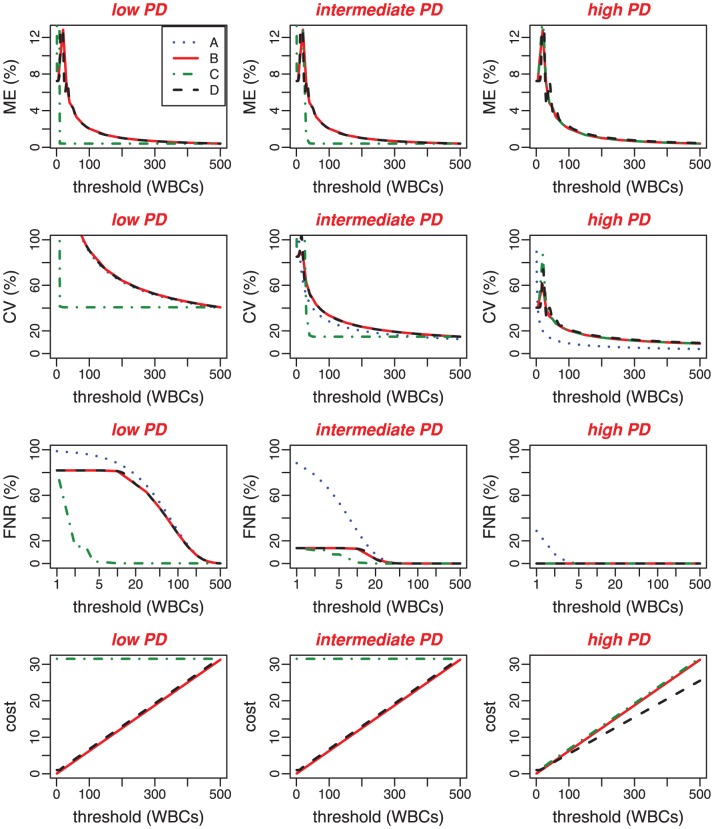
Statistical properties of PD estimators cut-offs according to threshold values for three PD levels : low (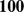 parasites/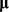
l), intermediate (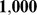 parasites/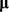
l) and high (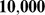 parasites/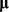
l). Variability measures (ME, CV, FNR) and cost are expressed as functions of the WBCs count (threshold) for the four methods (A, B, C, D). This graph gives the required number of WBCs for each method according to an expected amount of variability or cost, and favours a direct comparison between methods in terms of WBCs count. A logarithmic scale is used on the 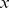-axis for FNR.

As Method A was shown to be unbiased, it was excluded from the ME analysis. As shown in Figure 5, Method B and Method D seem to have similar behaviors in terms of ME for low and intermediate parasitemias insofar as the two estimates are based on the leukocyte count in this density interval. For high parasitemias, Method B and Method C give the same results. The parasite count in Method C does not influence the accuracy of the method as long as parasites are numerous. For this reason, the two methods basically behave the same way.

To understand how the threshold values influence the variability of PD estimates, we plotted the CV according to threshold values. Figure 5 shows that the CV is highly sensitive to any variation of low thresholds (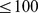). However, we see very few variations of the CV as threshold values increase (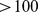). Both Methods B and D generate very close CV values for low and intermediate parasitemia levels. This result is expected since the number of WBCs seen is greater than the parasite number in the considered PD intervals. Hence, the two methods have the same stopping rules. For high parasitemias, Method B and Method C generate similar variability whereas Method A is significantly more precise than the other methods (B, C, D). However, Method A generates higher FNR for intermediate parasitemia than other methods when the count does not exceed 20 HPFs. For high PD levels, false negatives do not occur when the count exceeds 5 HPFs.

Figure 5 point out the high level of accuracy and precision performance of Method C for low and intermediate parasitemias. Thus, adding a supplementary stopping rule to the counting process and taking into account the parasite counts have enhanced the method performance, which raises questions regarding the repercussions in terms of cost-effectiveness. As shown in Figure 5, Method C is more expensive and time-consuming for low and intermediate parasitemias and requires constant cost (31.5 HPFs). As leukocytes are more present than parasites in the considered PD intervals the counting will be carried out until 500 WBCs are seen. Method A and B costs are density independent and increase linearly with threshold values. Method D outperformed the three other methods in terms of cost for high parasitemia levels.

### Variability of measurements at equal cost-effectiveness

**Figure 6:**
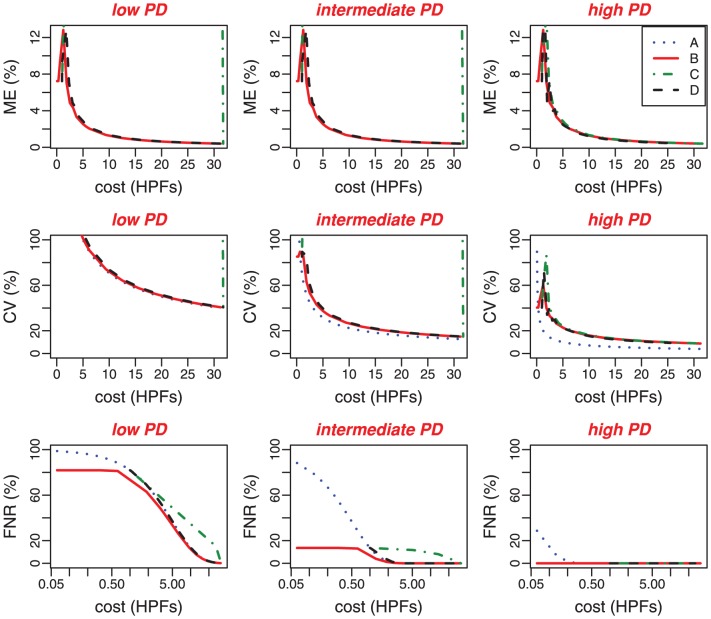
Statistical properties of PD estimators cut-offs according to methods cost for three PD levels : low (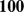 parasites/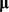
l), intermediate (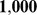 parasites/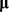
l) and high (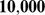 parasites/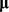
l). Variability measures (ME, CV, FNR) are expressed as functions of the cost (the number of HPFs needed to stop the counting) for the four methods (A, B, C, D). This graph gives the cost for each method according to an expected amount of variability, and favours a direct comparison between methods in terms of cost. A logarithmic scale is used on the 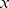-axis for FNR.

The duality between variability and cost illustrated in the previous section prompted a more detailed analysis of method performance differences at equal cost-effectiveness. In order to do this, we represent the variability measures as a function of cost. As shown in Figure 6, Methods B and D behave the same in terms of ME and CV for low and intermediate PD levels. For Method C, ME, CV and FNR are density independent for low and intermediate parasitemias. For high PD levels, Methods B, C and D present similar results for ME, CV and FNR. Method A has the lowest CV values but generates higher FNR.

### Methods comparison for standards threshold values

To identify both similarities and differences between the commonly used threshold-based counting techniques, we estimate ME, CV, FNR and cost as a percentage of PD according to three parasitemia levels (low, intermediate, high) for commonly used threshold values. We used 200 HPFs for Method A, 200 WBCs for Method B, 100 parasites and 200 WBCs for Method C and 500 WBCs or 500 parasites for Method D.

Table 1 shows that Method A is the most efficient method in terms of accuracy (ME) and precision (CV), but has an important cost (200 HPF). Conversely, Method B is less accurate and precise than Method A while much more cost-effective (12.5 HPFs). Method C and Method D present similar properties for low and intermediate parasitemia levels. In fact, Method C behaves as Method D with a fixed leukocyte count (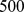 WBCs) due to the scanty presence of parasites. Hence, the mean error is density independent in these PD intervals. For high PD levels, Methods B and C behave the same. Due to the abundance of parasites, the enumeration is stopped when 200 WBCs are seen in both counting procedures. However, Method D is better suited to high parasitemia levels in terms of accuracy and precision compared to Methods B and C but results in up to a 2-fold increase in costs.

**Table 1:** Threshold-based counting techniques comparison for low (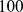 parasites/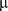
l), intermediate (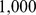 parasites/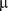
l) and high (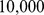 parasites/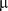
l) parasitemias.

Low parasitemia				
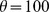 parasites/ 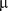 l				
Method	A	B	C	D
ME (%*_θ_*)	0.00	1.01	0.40	0.40
CV (%*_θ_*)	15.81	64.20	40.68	40.68
FNR (%)	0.00	7.56	0.18	0.18
Cost	200	12.50	31.75	31.75

Measures of variability (ME, CV, FNR) and cost-effectiveness of methods are compared for fixed threshold values : 200 HPFs for Method A, 200 WBCs for Method B, 100 parasites and 200 WBCs for Method C, and 500 WBCs or 500 parasites for Method D.

## Discussion

To the best of our knowledge, this is the first study of threshold-based counting technique performance using the theoretical properties of PD estimators. We considered four commonly used threshold-based counting techniques, and assessed the performances of these methods according to threshold values. These thresholds may be fixed or variable. We showed that adaptative methods are more efficient than the ones involving fixed threshold values. To define the theoretical properties of the estimators we hypothesized that the distribution of parasites within HPFs follows a Poisson distribution. We demonstrated that Method A estimator is unbiased and efficient. However, this estimator generates a high number of false negatives, especially for low parasitemia levels when the counting does not exceed few HPFs. Moreover, Method A is time-consuming. We showed that the ME of Method B is independent from PD, and only depends on the threshold value. This helps to handle the amount of bias with an appropriate choice of the WBC threshold value. We showed that adding a new parameter to the stopping rules (the number of parasites seen) implies more accuracy and precision without increasing the method's cost for low and intermediate parasitemias. Method B and Method D have similar behaviors for low and intermediate parasitemia levels while Method D is more accurate and precise in the considered PD intervals. For high parasitemia levels, Method B and Method C have similar behaviors and are more accurate and precise than Method D. However, for high parasitemias, Method D outperformed the three other methods in terms of cost. For each method, different threshold values may be fixed, which raises questions regarding the accuracy and reproducibility of these parasite counting techniques.

The importance of parasite density data reproducibility stems from the need for epidemiological interpretations to be based on solid evidence. However, variation of parasite density within a slide is expected even when prepared from a homogeneous sample [21]. The source and scale of measurement error (sample preparation, staining process, counting technique, microscopist performance) have been investigated. The notion of inter-rater reliability is a source of concern in this context. It refers to a metric for raters' consistency that measures the degree of agreement among raters. Many techniques were developed to measure inter-rater reliability. Some reports deal with the variability in the methods for detecting and counting parasites in thick smears. They attempt to evaluate the inter-rater reliability of malaria microscopy in epidemiological studies by looking at the variation of results due to the microscopist's reading. The variability of these methods has been assessed using statistical approaches [11], [12], [20], [21], [24]–[29]. These methods used several criteria to assess the inter-rater reliability and to quantify the degree of agreement between malaria slide density readings. For continuous data, Analysis of Variance (ANOVA) is the method of choice. Bland & Altman (1986) [24] plotted the differences in log-transformed data versus average in mean counts. They expanded on this idea by plotting the difference of each point, the mean difference, and the confidence limits on the vertical axis against the average of the two ratings on the horizontal axis. The resulting Bland & Altman plot [24] demonstrates not only the overall degree of agreement, but also whether the agreement is related to the underlying value of the item. For instance, two raters might nearly agree in estimating the size of small items, but disagree about larger ones. Alexander et al. (2010) [21] assessed agreement between replicate slide readings of malaria parasite density using as criterion the repeatability, that is to say the value below which the absolute difference between results may be expected to lie with a 95% probability [30]. This metric is linked to Bland & Altman limits of agreement [24]. It is half the distance between the upper and lower limits of agreements. For nominal data, the *kappa* coefficient of *Cohen* [31] and its many variants and the *Scott*'s *pi* [32] are the preferred statistics.

However, very few studies have examined the threshold-based counting techniques or evaluated the impact of the sampling error in endpoint measurements. In Nigeria, Dowling & Shute (1966) [12] showed that only 43% of infections in adults were detected by examining 200 fields, 61% by examining 600 fields and 70% by examining 1000 fields. In the Garki Project, Molineaux & Gramiccia (1980) [33] showed that the prevalence observed by the examination of 400 compared to 200 fields was increased by 10% for *P. falciparum*, by 24% for *P. malariae* and by 21% for *P. ovale*. Trape (1985) [11] compared the results of the examination of 100 and 200 fields of the thick film in 245 schoolchildren aged 6 to 16 from Linzolo (Congo). He concluded that the systemic examination of 200 oil immersion fields of the thick smear is the best compromise between the need for precision and rapidity. Prudhomme O'Meara et al. (2006) [34] showed empirically that counting beyond 200 WBCs may not significantly improve parasite density measurements.

In addition, the accuracy and consistency of these methods have been generally overlooked. There is no general agreement on the optimal method for estimating parasite density according to threshold values. Further experimental evidence is needed to determine which parasite counting technique is most accurate, reproducible, and efficient. Ultimately, the question is: to which extent would threshold values (specifically the number of WBCs counted and HPFs seen) influence the variability in parasite density estimates? However, there remains the issue of homogeneity. The distribution of the thickness of the smear and hence the distribution of parasites within the smear is not completely homogeneous [21]. Therefore, a proportion of the variability may be explained by this homogeneity factor.

To understand how the thresholds involved in parasite enumeration methods contribute to the magnitude of discrepancies in density determination, we studied their impact in variability measures generated by commonly used threshold-based counting techniques. We showed that estimators perform quite differently according to threshold values, and that an overall performance measure probably hides a lot of complexity in the behavior of each estimator. Another important aspect of this study is that we observed how estimators perform at different parasitemia levels, and how much the choice of threshold values may influence the performance of estimators relative to each parasitemia level. In summary, while all four estimators had some deficiencies, Method D outperformed all the other estimators for accuracy, precision measures and cost-effectiveness, and should therefore be seriously considered in future studies of comparative performance of PD estimators with field-collected data. In this paper, we explored the duality between cost-effectiveness and precision implied by estimation methods. An open question remains: To what extent is it possible to reduce methods' cost while staying accurate and precise in estimation measures?

Accurate estimation of PD is an important endpoint in epidemiological studies and clinical trials, both as a direct measure of the level of infection in a population and when defining parasitemia thresholds to diagnose malaria in case of fever episodes. Malaria PD estimates are also used to assess the development of naturally acquired immunity [35] and in malaria vaccine investigations [23], [36], [37]. Therefore, inaccurate estimation of PD can lead to patient mismanagement and public health misinformation [38].

However, two approaches must be distinguished. The first approach concerns clinical malaria diagnosis and two problems can be pointed out depending on whether the question concerns an individual or a population. At the individual level, recent studies have highlighted the massive problem of misdiagnosis in malaria endemic countries [39]–[41]. From a clinical point of view the question is to determine whether or not a person presenting fever suffers from malaria or not and, in that sense, the main problem is a false negative result. Here, if a measure is falsely negative, the patient will be miscategorized and incorrectly treated, and measurement errors can lead to poor patient outcome. Nevertheless, although the level of infection is considered as a controversial sign of potential severity [42], treatment and medical supervision must be immediately started even if the PD is not accurately determined.

From an epidemiological point of view (e.g. to determine the incidence or prevalence of clinical malaria in an area or in a population under close medical surveillance) clinical malaria is often considered as any case of fever or fever-related symptoms (headache, vomiting, subjective sensation of fever) associated with a *P. falciparum* parasite/leukocyte ratio higher than an age-dependent pyrogenic threshold of PD previously identified in the patient [43], [44]. In this case, a feverish individual harboring a PD under his age-specific threshold is not considered as a malaria case and will be monitored by the medical team involved in the study. In such a situation the accuracy of PD determination is obviously of great importance, not only for the patient but also for the outcomes and the conclusions of the study.

A second approach concerns the assessment of parasite density in epidemiological studies, when PD is used as the variable of interest, independently of clinical disease. For example, genetic epidemiology studies often focused on a mean level of *P. falciparum* infection during a follow-up period [43], [45]–[47]. Great care must also be taken in the analysis of parasite density estimates when parasite density is related to other explanatory variables, malariometric (e.g. parasite ratio, gametocyte ratio, mixed infection) or not (age, environmental or behavioral factors, medicine intake in clinical trials), when using statistical models as logistic regression and linear mixed effect models. In these cases as well as in population studies using a pyrogenic threshold to define clinical malaria, inaccurate estimates of parasite density might influence the parameters of associations between drug efficacy and the incidence of clinical malaria episodes in field trials [6], or between risk factors in epidemiological studies.

In further support of the arguments cited in this paper, empirical validation of the theoretical results is needed through a rereading experience conducted in the field. And toward a better understanding of threshold effects, we are interested in the study of the consequences of the quality of these estimators in models classically used and starting from these measures (mixed effects linear and logistic regression, generalized linear models, etc). These aspects of the problem are now under consideration.

## Supporting Information

File S1 **Supporting Information File.**
(PDF)
