# Supplementary material for: Statistical Properties of Parasite Density Estimators in Malaria
Source: PLoS One. 2013 Mar 14;8(3):e51987. doi: 10.1371/journal.pone.0051987 (PMC3597708; doi:10.1371/journal.pone.0051987)
Supplement: File S1 — Supporting Information File. (PDF) [file pone.0051987.s001.pdf]

Statistical properties of Parasite Density estimators in Malaria Supplementary Materials

Imen Hammami*,[1], Grégory Nuel[1], André Garcia[2]

[1]Department of Applied Mathematics (MAP5) UMR CNRS 8145,
Paris Descartes University, Paris, France.
[2]Department of Mother and Child in Relation to Tropical Infections UMR 216,
Institut de Recherche pour le Développement, Paris, France
*Corresponding author. E-mail:imen.hammami@parisdescartes.fr

1 Proof of efficiency of estimator $\widehat{\theta}_A$

- Let θ be the number of parasite per microliter of blood.
- $\widehat{\theta}_A$ denotes the estimator of θ for Method A.
- Let X be a random variable that represents the number of parasites per field.
- Let S_n be the sum of parasites in n consecutive fields.

Since $X_i \sim \mathcal{P}(\lambda_p)$ and X_i are iid, we have $S_n \sim \mathcal{P}(n\lambda_p)$. The number of parasite per field λ_p is then estimated by $\widehat{\lambda_p}$ where $\widehat{\lambda_p} = \frac{S_n}{n}$. Assuming the average amount of blood in each field as 0.002 μl, the PD is estimated by $\widehat{\theta}_A = \widehat{\lambda_p} \times 500$. Since $\mathbb{E}\left[\widehat{\theta}_A\right] = \theta$, $\widehat{\theta}_A$ is *unbiased*. In order to evaluate the efficiency of this estimator, the variance is to be compared against the *Fisher* Information $\mathrm{I}(\lambda_p)$. The variance of this unbiased estimator is bounded by the inverse of the $\mathrm{I}(\lambda_p)$ namely the *Cramer-Rao* Bound (CRB) as follows

$$var(\widehat{\lambda_p}) \geq \frac{1}{nI(\lambda_p)}$$

where

$$I(\lambda_p) = -\mathbb{E}_{\lambda_p}\left[\frac{\partial^2 \log L(X, \lambda_p)}{\partial^2 \lambda_p}\right]$$

The log likelihood function is defined by

$$\begin{aligned}
\mathcal{L}(x_i, \lambda_p) &= \log L(x_i, \lambda_p) \\
&= \log p(x_i, \lambda_p) \\
&= -\lambda_p - \log(x_i!) + x_i \log(\lambda_p) \\
\frac{\partial \log \mathcal{L}(x_i, \lambda_p)}{\partial \theta} &= -1 + \frac{x_i}{\lambda_p} \\
\frac{\partial^2 \log \mathcal{L}(x_1, \lambda_p)}{\partial^2 \theta} &= -\frac{x_i}{\lambda_p^2} \\
\mathbb{E}\left[\frac{\partial^2 \log \mathcal{L}(x_1, \lambda_p)}{\partial^2 \theta}\right] &= -\frac{1}{\lambda_p}
\end{aligned}$$

Then, $I(\lambda_p) = \frac{1}{\lambda_p}$, which gives $CRB = \frac{\lambda_p}{n}$.
The variance of the estimator is defined by

$$\begin{aligned}
var(\widehat{\lambda_p}) &= var(\frac{1}{n}\sum_{i=1}^{n} X_i) \\
&= \frac{1}{n^2}\sum_{i=1}^{n} var(X_i) \\
&= \frac{1}{n} var(X_i) \\
&= \frac{\lambda_p}{n}
\end{aligned}$$

Then, $var(\widehat{\lambda_p})$ reaches the *CRB*. Hence, $\widehat{\theta}_A$ is an *efficient* estimator of θ.

2 Validation study

To evaluate the performance of our models and to validate the theoretical results derived from estimators probability functions, we performed $10,000$ simulations. In each simulation step, we generate $1,000$ random drawings of θ, and we saved the sample ME, CV, FNR and cost in a vector. We compared simulated data to our results.

In the following, we present samples of the dataset used in our study. We give theoretical (*th*) and empirical (*emp*) values of variability measures (ME, CV, FNR) for methods A, B, C and D at three parasitemia levels (100 parasites/μl, 1,000 parasites/μl, 10,000 parasites/μl). Results are computed for 100, 200, 300, 400, 500 WBCs. θ denotes the number of parasite per microliter of blood, n denotes the number of fields, ℓ denotes the number of leukocytes, p denotes the number of parasites.

Table S1: Method A

θ (μl)	n	ℓ	$ME_{th}(\%\theta)$	$ME_{emp}(\%\theta)$	$CV_{th}(\%\theta)$	$CV_{emp}(\%\theta)$	$FNR_{th}(\%)$	$FNR_{emp}(\%)$
100	6.25	100	0.00	0.00	85.14	86.00 [84.45 87.55]	25.17	25.43 [24.72 26.15]
	12.50	200	0.00	0.00	60.28	60.88 [59.97 61.79]	6.38	6.45 [6.05 6.86]
	18.75	300	0.00	0.00	49.24	49.73 [49.05 50.41]	1.62	1.63 [1.42 1.84]
	25.00	400	0.00	0.00	42.65	43.08 [42.48 43.67]	0.41	0.41 [0.31 0.52]
	31.25	500	0.00	0.00	38.15	38.54 [38.04 39.04]	0.10	0.10 [0.05 0.15]
1,000	6.25	100	0.00	0.00	28.11	28.39 [28.04 28.74]	0.00	0.00
	12.50	200	0.00	0.00	19.90	20.10 [19.86 20.34]	0.00	0.00
	18.75	300	0.00	0.00	16.25	16.42 [16.23 16.61]	0.00	0.00
	25.00	400	0.00	0.00	14.08	14.22 [14.05 14.39]	0.00	0.00
	31.25	500	0.00	0.00	12.60	12.72 [12.57 12.87]	0.00	0.00
10,000	6.25	100	0.00	0.00	8.92	9.01 [8.90 9.12]	0.00	0.00
	12.50	200	0.00	0.00	6.32	6.38 [6.30 6.46]	0.00	0.00
	18.75	300	0.00	0.00	5.16	5.21 [5.15 5.27]	0.00	0.00
	25.00	400	0.00	0.00	4.47	4.51 [4.46 4.56]	0.00	0.00
	31.25	500	0.00	0.00	4.00	4.04 [3.99 4.09]	0.00	0.00

Table S2: Method B

θ (μl)	ℓ	$ME_{th}(\%\theta)$	$ME_{emp}(\%\theta)$	$CV_{th}(\%\theta)$	$CV_{emp}(\%\theta)$	$FNR_{th}(\%)$	$FNR_{emp}(\%)$
100	100	2.03	2.05 [1.15 2.95]	90.58	91.48 [89.74 93.22]	26.17	26.43 [25.71 27.16]
	200	1.01	1.02 [0.34 1.70]	64.20	64.84 [63.78 65.89]	7.56	7.63 [7.20 8.07]
	300	0.67	0.68 [0.15 1.21]	52.47	53.00 [52.22 53.78]	2.18	2.20 [1.96 2.44]
	400	0.50	0.51 [0.05 0.97]	45.47	45.92 [45.27 46.57]	0.63	0.64 [0.51 0.77]
	500	0.40	0.41 [0.00 0.82]	40.68	41.09 [40.53 41.64]	0.18	0.18 [0.11 0.25]
1,000	100	2.03	2.05 [1.72 2.38]	33.42	33.76 [33.33 34.19]	0.00	0.00
	200	1.01	1.02 [0.79 1.25]	23.53	23.77 [23.48 24.06]	0.00	0.00
	300	0.67	0.68 [0.48 0.88]	19.19	19.38 [19.15 19.61]	0.00	0.00
	400	0.50	0.51 [0.34 0.68]	16.61	16.77 [16.56 16.98]	0.00	0.00
	500	0.40	0.41 [0.26 0.56]	14.85	15.00 [14.82 15.18]	0.00	0.00
10,000	100	2.03	2.05 [1.84 2.26]	20.21	20.41 [20.17 20.65]	0.00	0.00
	200	1.01	1.02 [0.88 1.16]	14.03	14.17 [14.00 14.34]	0.00	0.00
	300	0.67	0.68 [0.57 0.79]	11.39	11.50 [11.36 11.64]	0.00	0.00
	400	0.50	0.51 [0.41 0.61]	9.83	9.93 [9.81 10.05]	0.00	0.00
	500	0.40	0.41 [0.32 0.50]	8.78	8.87 [8.76 8.97]	0.00	0.00

Table S3: Method C

θ (μl)	p	ℓ	$ME_{th}(\%\theta)$	$ME_{emp}(\%\theta)$	$CV_{th}(\%\theta)$	$CV_{emp}(\%\theta)$	$FNR_{th}(\%)$	$FNR_{emp}(\%)$
100	50	100	0.40	0.41 [0.01 0.81]	40.68	41.09 [40.53 41.65]	0.18	0.18 [0.11 0.25]
	100	200	0.40	0.41 [0.01 0.81]	40.68	41.09 [40.53 41.65]	0.18	0.18 [0.11 0.25]
	150	300	0.40	0.41 [0.01 0.81]	40.68	41.09 [40.53 41.65]	0.18	0.18 [0.11 0.25]
	200	400	0.40	0.41 [0.01 0.81]	40.68	41.09 [40.53 41.65]	0.18	0.18 [0.11 0.25]
	250	500	0.40	0.41 [0.01 0.81]	40.68	41.09 [40.53 41.65]	0.18	0.18 [0.11 0.25]
1,000	50	100	0.40	0.41 [0.27 0.55]	14.85	15.00 [14.82 15.17]	0.00	0.00
	100	200	0.40	0.41 [0.27 0.55]	14.85	15.00 [14.82 15.17]	0.00	0.00
	150	300	0.40	0.41 [0.27 0.55]	14.85	15.00 [14.82 15.17]	0.00	0.00
	200	400	0.40	0.41 [0.27 0.55]	14.85	15.00 [14.82 15.17]	0.00	0.00
	250	500	0.40	0.41 [0.27 0.55]	14.85	15.00 [14.82 15.17]	0.00	0.00
10,000	50	100	2.03	2.05 [1.85 2.25]	20.21	20.41 [20.17 20.64]	0.00	0.00
	100	200	1.01	1.02 [0.88 1.16]	14.03	14.17 [14.00 14.34]	0.00	0.00
	150	300	0.67	0.68 [0.57 0.79]	11.39	11.50 [11.37 11.63]	0.00	0.00
	200	400	0.50	0.51 [0.41 0.61]	9.83	9.93 [9.82 10.04]	0.00	0.00
	250	500	0.40	0.41 [0.32 0.50]	8.78	8.87 [8.77 8.97]	0.00	0.00

Table S4: Method D

θ (μl)	$\ell = p$	$ME_{th}(\%\theta)$	$ME_{emp}(\%\theta)$	$CV_{th}(\%\theta)$	$CV_{emp}(\%\theta)$	$FNR_{th}(\%)$	$FNR_{emp}(\%)$
100	100	2.03	2.05 [1.14 2.96]	90.58	91.48 [89.73 93.23]	26.17	26.43 [25.71 27.16]
	200	1.01	1.02 [0.38 1.66]	64.20	64.84 [63.80 65.88]	7.56	7.63 [7.20 8.07]
	300	0.67	0.68 [0.16 1.20]	52.47	53.00 [52.23 53.77]	2.18	2.20 [1.96 2.44]
	400	0.50	0.51 [0.06 0.96]	45.47	45.92 [45.28 46.56]	0.63	0.64 [0.51 0.77]
	500	0.40	0.41 [0.02 0.80]	40.68	41.09 [40.51 41.66]	0.18	0.18 [0.11 0.25]
1,000	100	2.03	2.05 [1.72 2.38]	33.42	33.76 [33.33 34.19]	0.00	0.00
	200	1.01	1.02 [0.79 1.25]	23.53	23.77 [23.48 24.06]	0.00	0.00
	300	0.67	0.68 [0.49 0.87]	19.19	19.38 [19.15 19.61]	0.00	0.00
	400	0.50	0.51 [0.35 0.67]	16.61	16.77 [16.56 16.98]	0.00	0.00
	500	0.40	0.41 [0.26 0.56]	14.85	15.00 [14.82 15.18]	0.00	0.00
10,000	100	2.29	2.31 [2.10 2.52]	21.45	21.67 [21.41 21.93]	0.00	0.00
	200	1.14	1.15 [1.01 1.29]	14.89	15.04 [14.86 15.21]	0.00	0.00
	300	0.76	0.77 [0.66 0.88]	12.09	12.21 [12.06 12.36]	0.00	0.00
	400	0.57	0.57 [0.47 0.67]	10.44	10.54 [10.41 10.66]	0.00	0.00
	500	0.45	0.46 [0.37 0.55]	9.31	9.41 [9.30 9.52]	0.00	0.00
